# Safety and Efficacy of Anti-B-cell Maturation Antigen (Anti-BCMA) Bispecific Antibodies for Relapsed/Refractory Multiple Myeloma: A Systematic Review of Clinical Trials

**DOI:** 10.7759/cureus.98368

**Published:** 2025-12-03

**Authors:** Asad Ali Khan, Mansoor Rahman, Inshal Jawed, Muhammad Sajjad, Muhammad Musa Khan

**Affiliations:** 1 Internal Medicine, University Hospitals Birmingham NHS Foundation Trust, Birmingham, GBR; 2 Internal Medicine, Hamilton Medical Center, Dalton, USA; 3 Internal Medicine, Khyber Medical College, Peshawar, Peshawar, PAK; 4 Dermatology, Dow University of Health Sciences, Karachi, PAK; 5 Internal Medicine, Hayatabad Medical Complex Peshawar, Peshawar, PAK; 6 Acute and General Medicine, Good Hope Hospital, Birmingham, GBR

**Keywords:** antibody-drug conjugates (adcs), b-cell maturation antigen (bcma), bcma×cd3 bispecific antibody, bispecific antibodies, chimeric antigen receptor (car) t-cell therapy, relapsed and refractory multiple myeloma

## Abstract

Relapsed/refractory multiple myeloma (RRMM) remains a challenging condition with a need for more effective treatment options. There are ongoing clinical trials analyzing the effects of anti-B-cell maturation antigen (BCMA) bispecific antibodies (Abs) against RRMM with early, promising results. This study aims to systematically evaluate the safety and efficacy of anti-BCMA bispecific antibodies in patients with RRMM.

PubMed, Embase, Web of Science, and the American Society of Hematology (ASH) website were searched for published evidence on the safety and efficacy of anti-BCMA bispecific Abs. Screening was performed using original clinical trials published in English, RRMM, and anti-BCMA-CD3 bispecific Abs as our inclusion criteria.

Our search yielded a total of 2211 articles. After screening, we found 11 relevant clinical trials (five phase I, one phase I/II, two phase Ib, two phase II, one phase III). Across the trials, 910 patients with ages ranging from 32 to 82 years were analyzed. A majority of patients were exposed to and/or refractory to triple-class and penta-drugs (prior therapies ranged from 1 to 25). AMG-420, AMG-701, elranatamab, REGN5458, teclistamab (Tec), alnuctamab (ALNUC), and ABBV-383 are the seven anti-BCMA-CD3 bispecific Abs currently being assessed in clinical trials as monotherapy and in combination with immunomodulators/proteasome inhibitors against RRMM. Across the included trials, elranatamab demonstrated overall response rates (ORRs) of 61-64%, while teclistamab ranged from 40% to 78% depending on the regimen. ALNUC and ABBV-383 achieved ORRs of 51% and 57%, respectively. Among patients previously exposed to and/or refractory to anti-BCMA therapies (including antibody-drug conjugates and CAR-T cell therapy), ORRs were 54% for elranatamab and 40% for teclistamab. Incidence of cytokine release syndrome (CRS) or immune effector cell-associated neurotoxicity syndrome (ICANS) was low with subcutaneous (SC) administration of tec, elranatamab, and ALNUC. Across the studies, no death was reported due to CRS, although it led to treatment discontinuation in one patient in AMG-420, two patients in AMG-701, and dose reduction in three patients in ABBV-383 trials.

Our analysis of the trials revealed that bispecific Abs showed efficacy in RRMM, with CRS and hematologic toxicities being the most common adverse events, mostly low-grade and manageable. Based on the promising efficacy and safety of BCMA targeting bispecific Abs, these drugs are emerging as a new therapeutic option for patients with advanced and RRMM.

## Introduction and background

Multiple myeloma (MM) belongs to hematologic malignancies caused by clonal proliferation and overproduction of monoclonal immunoglobulins, causing several clinical manifestations. It is the second most prevalent blood disorder worldwide, primarily affecting older adults, and is associated with high morbidity due to end-organ dysfunction. African American populations are estimated to have the disease about twice as frequently as European Americans do [1,2].

The survival and prognosis are evaluated based on the level of disease, cytogenetic anomalies, patient factors, and previous treatments received [3]. In the last 10 years, immunomodulatory agents followed by proteasome inhibitors, and the antibodies of monoclonal origin have improved patient outcomes in MM when typically administered together or in conjunction with the autologous stem cell transplantation [4]. Nevertheless, patients with high-risk cytogenetics, i.e., deletion or translocations, or patients suffering from long-term disease frequently relapse early or develop refractory disease (RRMM), whereas low-risk patients can also develop resistance over time [5,6]. RRMM remains a significant concern. Thus, newer therapeutic strategies are required to control its expansion and deteriorating effects.

B-cell maturation antigen (BCMA) is a cell surface glycoprotein receptor found on the mature and immature plasma cell. Anti-BCMA agents are engineered proteins that simultaneously bind to BCMA on myeloma cells and CD3 on T-cells, directing the patient's own T-cells to kill the cancer cells. These agents have created a new hope for RRMM patients and are proving to be ground-breaking. This group includes bispecific antibodies (bsAbs), chimeric antigen receptor (CAR) T cells, and antibody drug conjugates (ADCs) [7-9]. Clinical trials are underway in these modalities to treat RRMM. Anti-BCMA-CD3 bsAbs have shown significant activity in early-phase research [10,11].

Our systematic review will review the existing data on the effectiveness and safety of anti-BCMA BsAbs when used in the treatment of RRMM, and outline their clinical role and their possible future contribution.

## Review

Methods

Data Sources and Searches

This study was completed in line with the Preferred Reporting Items for Systematic Reviews and Meta-Analyses (PRISMA) statement [12]. Pubmed, Embase, and Web of Science were searched for evidence on 18 February 2025, using the following medical subject heading (MeSH) terms: “multiple myeloma’’ AND ‘’bispecific antibodies," and related keywords. The search strategy we used was ("Antibodies, Bispecific"[Mesh]) AND ("Multiple Myeloma"[Mesh]). The related keywords were plasma cell malignancy, bispecific monoclonal antibody (BsMAb), and BsAb.

Study Selection and Eligibility Criteria

The following criteria were used for study selection: clinical trials as the study design; inclusion of patients with relapsed/refractory multiple myeloma (RRMM) or those intolerant to currently established therapies; use of anti-BCMA-CD3 bsAbs either as monotherapy or in combination; and evaluation of both safety and efficacy outcomes.

Based on the Population, Intervention, Comparison, Outcomes (PICO) framework, the population (P) consisted of adults aged ≥18 years with RRMM; the intervention (I) included bsAbs targeting BCMA (such as teclistamab (TEC), elranatamab, talquetamab, and other eligible agents); the comparison (C) was standard therapy or none, including both single-arm and comparative studies; and outcomes (O) encompassed efficacy - overall response rate (ORR), complete response (CR), stringent complete response (sCR), very good partial response (VGPR), duration of response (DoR), progression-free survival (PFS), and overall survival (OS) - as well as safety endpoints, including cytokine release syndrome (CRS), immune effector cell-associated neurotoxicity syndrome (ICANS), grade ≥3 adverse events (AEs), serious infections, treatment discontinuations, and deaths.

Data Extraction

**Figure 1:**
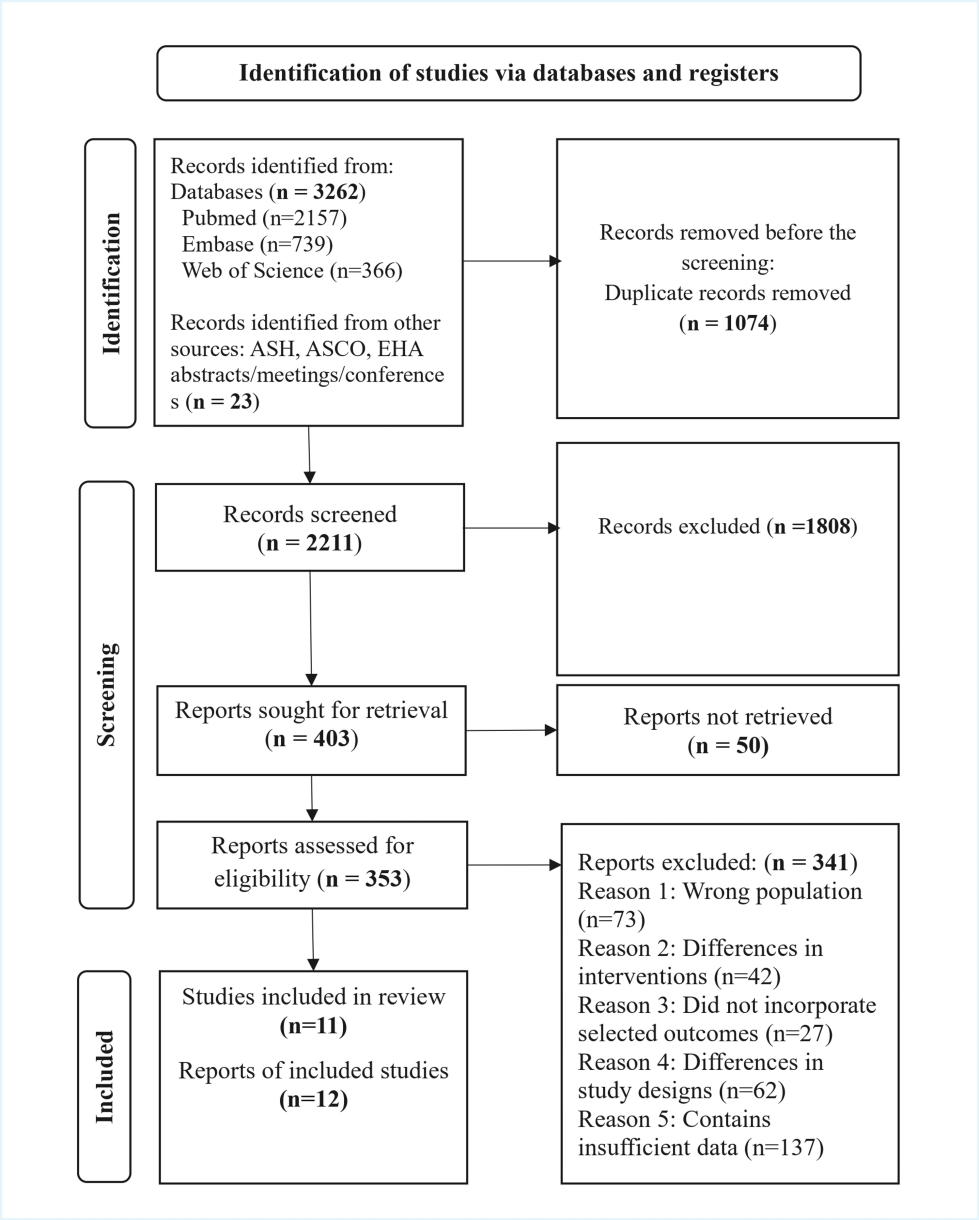
PRISMA flow diagram: Identification of studies via databases and registers. PRISMA: Preferred Reporting Items for Systematic Reviews and Meta-Analyses, ASH: American Society of Hematology, ASCO: American Society of Clinical Oncology, EHA: European Hematology Association

Search results were screened by two independent reviewers (IJ and MS). Our database search identified 3262 records and an additional 23 records from other sources (American Society of Hematology (ASH), the American Society of Clinical Oncology (ASCO), and the European Hematology Association (EHA) abstracts/conferences/meetings), giving a total of 3285 records. After removing 1074 duplicates, 2211 records remained for title and abstract screening. Of these, 1808 were excluded as irrelevant (predominantly due to non-eligible populations). We retrieved 403 reports for full-text assessment, of which 50 could not be obtained. A total of 353 reports were assessed for eligibility; 341 were excluded for reasons including wrong population (n=73), differences in intervention (n=42), not incorporating selected outcomes (n=27), differences in study designs (n=62), or because of insufficient data (n=137). Finally, 11 studies and 12 reports of the included studies were selected that met the inclusion criteria and were selected for the qualitative synthesis. Any conflicts that were found regarding the inclusion of a study were resolved after discussing and reviewing with a third reviewer (MR) (Figure 1).

For each study the following data was extracted into a pre-specified table: general data (trial registration number, study design, year of publication, current status of study); baseline characteristics of participants (number, median age, sex); features of disease (number of therapies used, high risk cytogenetics, Revised International Staging System (RISS) stage, Eastern Cooperative Oncology Group Performance (ECOG) status, prior use of anti-BCMA therapy, history of autologous stem cell transplant); efficacy outcomes using International Myeloma Working Group (IMWG) criteria, ORR, CR, sCR, partial response (PR), VGPR, minimal residual disease negative (MRD) status and PFS [13]; and safety outcomes (hematological and non-hematological toxicity based on Common Terminology Criteria for Adverse Events (CTCAE) v5.0 [14], CRS and ICANS based on Lee et al. [15] or American Society for Transplantation and Cellular Therapy (ASTCT) criteria [16]).

Quality Evaluation

The methodological quality of studies was evaluated using the National Institutes of Health (NIH) quality assessment tool for intervention studies [17]. It is based on the following categories: study objectives (demonstration of study aims and inclusion criteria); subject selection (selection of candidates fitting the criteria of inclusion, consistent delivery of intervention, sufficient sample size); comparability of subjects (inclusion of all candidates meeting the eligibility criteria); and clinical outcome (pre-specification of the outcomes assessed, blinding of the outcomes assessors, percentage of participants lost to follow up, statistical analysis of outcomes).

Based on the NIH quality assessment tool, among the studies from the 11 trials, nine were rated as good quality and two as fair quality. Most studies clearly described their objectives, inclusion criteria, interventions, and outcome measures. However, blinding of outcome assessors was not reported in seven trials, and randomization was absent in all early-phase single-arm designs, contributing to a moderate risk of bias. Sample sizes were generally small, and follow-up duration was short in most studies, limiting generalizability. Despite these limitations, the consistency of findings across studies shows that the observed efficacy and safety trends were robust.

Results

A total of 2211 citations were screened, out of which 11 relevant clinical trials were found [18-29]. Across the 11 trials, a total of 910 patients were included. The calculated mean age was 50.04 years (assuming a normal distribution), and the number of prior lines of therapy ranged from one to 25. AMG420, AMG701, elranatamab, Linvoseltamab (REGN5458), TEC, alnuctamab (ALNUC), and Etentamig (ABV-383) were the anti-BCMA-CD3 bsAbs evaluated against RRMM as monotherapy and in combination regimens.

AMG-420

In the first-in-human phase I study (NCT03836053), AMG-420 was given to 42 patients with RRMM (median age: 65 years; 64% male; median of prior line of therapies: 5). Median duration of the disease was 5.2 years; 33% had high-risk cytogenetics, 29% had prior daratumumab (DARA), 10% prior elotuzumab, and 86% prior autologous stem cell transplantation (SCT) (Table 1). AMG-420 was delivered IV for four weeks of a six-week cycle (dose: 0.2-800 μg/day). Overall, three completed 10 cycles, two remained on treatment, 25 discontinued for progression, four died, and one withdrew consent. Median treatment exposure was one cycle; however, responders received a median of seven cycles (Table 2) [18].

**Table 1:** Baseline characteristics of the included studies CT No: clinical trial number, LOT: line of therapies, ECOG: Eastern Cooperative Oncology Group Performance status, RISS: Revised International Staging System stage, MC NRCT: multicenter non-randomized controlled trial, IMiDs: immunomodulatory drugs, PI: proteosome inhibitors, Triple-ref: triple-refractory (refractory to PIs, IMiDs and CD38 monoclonal antibodies), Penta-ref: refractory to all five major treatment classes, i.e., two IMiDs, two PIs, and one CD38 monoclonal antibody (mAb), quad-ref: refractory to four major drug classes including PIs, IMiDs, corticosteroids and CD38 monoclonal antibodies

CT no./drug(s)	Study type	Sample size	Median age (years)	Median prior LOT	Disease refractory (%)	Transplant history (%)	ECOG (0/1/2-3%)	R-ISS (I/II/III%)	Median follow-up (months)
NCT0383605/AMG-420 [18]	Phase-1, MC NRCT	42	65	5	IMiDs 48%, PI 38%, both 36%	86%	57/40/2	-/-/-	-
NCT03287908/AMG-701 [19]	Phase-1, MC NRCT	75	63	6	68% triple-ref	83%	-	-/-/-	1.7
NCT03269136/Elranatamab [20]	Phase-1, MC NRCT	55	64	5	91% triple-ref	69%	-	-/-/-	12
NCT04649359/Elranatamab [21]	Phase-2, MC NRCT	123	68	5	96.7% triple-ref, 42.3% penta-ref	-	36.6/57.7/5.7	-/15.4/-	6.8
NCT05020236/Elranatamab, Daratumumab [22]	Phase-3, MC RCT	28	68	5	18% triple-ref	71%	-	-/-/-	NA
NCT03761108/REGN5458 [23]	Phase-1/2, MC NRCT	73	64	5	89% triple-ref, 70% quad-ref, 38% penta-ref	64.40%	-	15/57.5/23.3	3
NCT03145181/Teclistamab [24,25]	Phase-1, MC NRCT (MajesTEC-1)	157 (40 in RP2D)	63	6	Triple-class 82%, penta-drug 39%, last line 90%	85%	39/61/-	48/31/21	8.8-16.6 (6.1 in RP2D)
	Phase-1/2, MC NRCT	38	63.5	6	66% anti-BCMA, 84% last LOT	-	-	-/-/-	6.9
NCT04722146/SC Teclistamab + Daratumumab + Lenalidomide [26]	Phase-1b, MC NRCT (MajesTEC-2)	32	62	2	N/A	-	-	-/-/-	5.8
NCT04108195/Teclistamab + Daratumumab [27]	Phase-1b, MC NRCT (TRIMM-2)	46	67	6	74% triple-ref, 73% penta-ref	-	-	-/-/-	7.2
NCT03486067/CC-93269/BMS-986349/Alnuctamab/ALNUC [28]	Phase-1, MC NRCT	117	63	4	SC: 62% triple-ref, 21% penta-ref	-	-	-/-/-	4.3
NCT03933735/TNB-383B/ABBV-383 [29]	Phase-1, MC NRCT	124	68	5	Double-ref 84%, triple-ref 82%, penta-ref 35%	-	7/32/57	-/9/15/19/31	10.8

**Table 2:** Study design and intervention RP2D: recommended phase 2 dose, MTD: maximum tolerated dose, DLT: dose-limiting toxicity, CTCAE: common terminology criteria for adverse events, IMWG: International Myeloma Working Group, ORR: overall response rate, BICR: Blinded Independent Central Review, Tec: teclistamab, Dara: daratumumab, Len: lenalidomide, SC: subcutaneous, QW: once weekly, Q2W: once every two weeks, Q3W: once every three weeks

CT no./drug(s)	Drug regimen with dosing scheme	Recommended phase 2 dose RP2D	Dosing cohorts	Step-up dosage	Primary end-point of the study	Secondary end-point of the study
NCT0383605/AMG-420 [18]	IV for 4 weeks followed by 2 weeks break in 6-week cycles	400μg/day as MTD	Single and 3-6 patients cohorts at 0.2-800μg/day dosage	-	To find MTD and DLT for AMG420	To assess efficacy by CTCAE V4.0, anti-tumor effects and PK
NCT03287908/AMG-701 [19]	Weekly for 4 weeks	-	5-45 μg (1 patient in each), 0.14-1.2 mg (3-4 patients each), 1.6-12 mg (3-10 patients each)	0.8mg once IV	-	-
NCT03269136/Elranatamab [20]	sc 80-1000µg/kg QW or Q2W	1000µg/kg or 76mg	Patients in different cohorts received SC ≥215μg/kg	Single priming dose 600µg/kg	toxicity and safety of Elranatamab	Antimyeloma activity as per IMWG
NCT04649359/Elranatamab [21]	1000µg/kg or 76mg QW	1000µg/kg or 76mg	Cohort A (no antiBCMA use prior), cohort B(prior antiBCMA use, to be recruited)	2 step-up priming doses (12mg and 32 mg)	ORR by BICR	To assess safety
NCT05020236/Elranatamab, Daratumumab [22]	SC elranatamab + SC DARA in part 1	To be determined	In part 1, combination of Elranatamab (two different doses given sequentially) + Dara (dose as per guidelines) SC QW in 28-d cycles for C6 then Q2W for 2 or more months if PR or better with the previous dose. 1st combination dose administered at D8 of C1.	2 step-up doses in week 1 of C1	DLT	-
NCT03761108/REGN5458 [23]	IV 3-800 mg REGN5458 monotherapy following a modified 3 + 3 dose-escalation design (4 + 3)	-	IV 3-800mg 16 QW REGN5458 followed by q2w dosing until disease progression	Dose escalation following modified 3 + 3 dose-escalation design (4 + 3)	safety, tolerability, DLT, and RP2DR	Clinical activity as per IMWG
NCT03145181/Teclistamab [24,25]	IV 0.3-19.2 μg/kg Q2W, IV 19.2-720 μg/kg QW, SC 80-3000 μg/kg	SC 1500 μg/kg QW	IV 0·3-19·2 μg/kg Q2W n=12, IV 19·2-720 μg/kg n=72, SC 80-3000 μg/kg n=73	Full doses of 38·4 μg/kg or higher	Part 1 = frequency and type of dose-limiting toxicities, part 2 = incidence, severity of adverse events, serious adverse events, lab values	ORR, duration of response, time to response, pharmacokinetic parameters, pharmacodynamic markers, and anti-Tec antibodies
	SC Tec 1.5 mg/kg QW (step-up doses of 0.06 and 0.3 mg/kg)	-	1 Cohort, SC Tec 1.5 mg/kg QW (step-up doses of 0.06 and 0.3 mg/kg)	0.06 and 0.3 mg/kg	ORR per IMWG 2016 criteria	-
NCT04722146/SC Teclistamab + Daratumumab + Lenalidomide [26]	SC Tec+Dara+Len (0.72 or 1.5mg/kg,1800mg and 25mg). Dosing schedule NA	-	two-cohorts, Tec (0.72mg/kg) and 1.5mg/kg with fixed dose of Dara 1800mg and Len 25mg QW	-	-	-
NCT04108195/Teclistamab + Daratumumab [27]	SC Dara 1800mg and Tec 1.5-3mg/kg Q2 or Q2W	To be determined	SC Dara 1800mg and Tec 1.5mg/kg QW, SC Dara 1800mg and Tec 3.0mg/kg QW, SC Dara 1800mg and Tec 3mg/kg Q2W	-	Tec RP2D and safety of the combination	-
NCT03486067/CC-93269/BMS-986349/Alnuctamab/ALNUC [28]	In IV cohorts (0.15mg-10mg), QW for C1-3 then Q2W for C4-6 followed by Q4W onward. In SC cohorts (10-60mg) in C1 (D1, and 4th of W1 then QW), weekly in C2-3, biweekly C4-6, every 4W C7 onwards	-	IV and SC in 28-d cycles	Given in stage 2 of esc phase in IV cohorts. In SC cohorts, 3 and 6mg on D1 and D4 of C1 given	Finding MTD, NTD and RP2D for Alnuctamab	Clinical activity
NCT03933735/TNB-383B/ABBV-383 [29]	IV Q3W. 14 dose levels ranging from 0.025mg to 120mg in dose escalation phase and 60mg in dose expansion phase were evaluated	-	-	-	RP2D and MTD (safety and tolerability) of ABBV-383	Assess efficacy of ABBV-383

Efficacy became apparent at ≥100 μg/day. Across the study, ORR was 31% (n=13/42), including six MRD-negative CRs (one at 200 μg/day, five at 400 μg/day), three additional CRs (at 6.5, 100, and 800 μg/day), two VGPRs (at 400 and 800 μg/day), and two PRs (at 50 and 400 μg/day). At 400 μg/day, ORR was 70% (7/10; five MRD-CR, one VGPR, one PR). One month was the median response time, with most responders achieving first response in cycle one. Responses lasted a median of 8.4 months (range 2.5-≥15.5), with several exceeding one year; seven of 13 responses were ongoing at cutoff. MRD-negative responses lasted a median of 9.6 months. Responses were observed across all cytogenetic risk categories (Table 3) [18].

**Table 3:** Efficacy outcomes of the included studies ORR: Overall response rate, SCR: stringent complete response, CR: complete response, PR: partial response, VGPR: very good partial response, MRD: minimal residual disease, PFS: progression-free survival, Med DoR: median duration of response, SC: subcutaneous, QW: once weekly

CT no./drug(s)	ORR	sCR	CR	PR	VGPR	MRD- status	PFS	Med DoR	Median months to response
NCT0383605/AMG-420 [18]	31% (N=13/42)	-	21.42% (9/42)	4.8% 2/42	4.8% (2/42)	14.3% 6/42	-	Overall 8.4 months (2.5 to ≥15.5). For MRD- 9.6 months (2.8 to ≥12.8 months)	1
NCT03287908/AMG-701 [19]	36% (n=17/69)	5.8% (4/69)	1.5% (1/69)	8.7% (6/69)	8.7% (6/69)	5.8% (4/69)	-	3.8 (1.9-7.4) months	1
NCT03269136/Elranatamab [20]	64% (35/55) with 95% CI 50-75%	-	38% (21/55)	-	56% (31/55) VGPR or better	100% (12/12)	59% at 1-year	17.1 months (95% CI 10.6-NE) as per KM estimation	1.2
NCT04649359/Elranatamab [21]	61%	-	-	-	-	-	90.4% at 6 months	Not reached yet	1.2
NCT05020236/Elranatamab, Daratumumab [22]	-	-	-	-	-	-	-	NA	1
NCT03761108/REGN5458 [23]	50.6% (37/73)	-	43.2% (n=16/37)	-	86.5% (32/37)	-	-	Not reached	-
NCT03145181/Teclistamab [24,25]	70% 32/46 (IV 270 μg/kg + 720 μg/kg and SC 720 μg/kg + 3000 μg/kg QW), 65% 26/40 (SC 1500 μg/kg QW)	9% 4/46 (IV 270 μg/kg + 720 μg/kg and SC 720 μg/kg + 3000 μg/kg QW), 18% 7/40 (SC 1500 μg/kg QW)	28% 13/46, 23% 9/40	2% 1/46, 8% 3/40	30% 14/46, 18% 7/40	69% (18/26)	67% at recommended Phase 2 dose	Not reached	1
	40%	-	20%	-	-	-	-	not reached	1.2
NCT04722146/SC Teclistamab + Daratumumab + Lenalidomide [26]	89.65% (n=26/29). 100% (13/13) at 0.72 mg/kg cohort	-	-	-	92.3% (12/13) for 0.72mg/kg cohort	-	-	NA	1
NCT04108195/Teclistamab + Daratumumab [27]	78% (29/37)	-	24.3% (9/37)	-	73% (27)	-	-	N/A	1
NCT03486067/CC-93269/BMS-986349/Alnuctamab/ALNUC [28]	IV cohort, 39% (n=27/70), SC cohorts, 53%	In SC cohort 17% (7/41), at ≥30mg 23% (3/13)	-	24% (n=10/41), 46% (6/13) ≥30mg	In SC cohort, overall 10% (4/41), 8% (1/13) at ≥30mg	80% (16/20) in 55 SC cohort	In IV cohort, 3.1 months. In SC cohorts, Med PFS 13.3 wks (95% CI 8.1-23.9)	In IV cohort, 33.6 months. In SC cohorts, 146.1 wks (95% CI 40.6-NR)	-
NCT03933735/TNB-383B/ABBV-383 [29]	57% (69/122)	17% (21/122)	11% (14/122)	14% (17/122)	14% (17/122)	73% (8/11)	Median PFS for all patients 10.4 (5.0-19.2) months	Estimated 6/12 months DoR for all responding patients (n=69) 76.3% (95% CI 63.2 to 85.3) 67.2% (95% CI 52.6 to 78.2)	-

Dose-limiting toxicities (DLTs) occurred at 800 μg/day (two out of three patients). Grade 3 CRS (n=1) and grade 3 peripheral polyneuropathy (n=2) were dose-limiting; both neuropathy cases improved to baseline within two to three months. CRS occurred in 38% (n=16/42), mostly grades 1-2, and was managed supportively; one case required tocilizumab. Infections were reported in 33% (n=14/42), including eight grade 3 and two grade 5 (aspergillosis/influenza, adenovirus). Serious adverse effects were recorded in 48% (n=20), among them, the most common were infections (n=14) and polyneuropathy (n=2). Additional AEs included transient aspartate transaminase (AST)/alanine transaminase (ALT) elevations in five patients, usually coincident with CRS. No anti-drug antibodies were detected (Table 4) [18]. AMG-420, therefore, demonstrated meaningful anti-myeloma activity at 400 μg/day with durable MRD-negative CRs, but dose-limiting neuropathy and infection risk limited tolerability at higher doses [18].

**Table 4:** Safety profile of the patients in the included studies CRS: cytokine release syndrome, ICANS: Immune Effector Cell-Associated Neurotoxicity Syndrome, DLT: dose-limiting toxicity, d/c: discontinued, CTCAE: Common Terminology Criteria for Adverse Events, ASTCT: American Society for Transplantation and Cellular Therapy, Esc: escalation cohort (0.025-120 mg), Exp: expansion cohort (60 mg), AE: adverse effect, PD: progression of disease

CT no./drug(s)	Overall hematological, n (%)	Hematological G 3/4	Non-hematological G 3/4	No. of patients discontinued treatment	CRS overall and G 3/4	CRS managed with standard meds or tocilizumab (no of patients with grade of CRS)	No of patients died of CRS or discontinued treatment due to CRS	ICANS/neurotoxicity, overall and G 3/4
NCT0383605/AMG-420 [18]	-	-	19% (n=8/42) G3 infections, 4.7% had G5 infections (n=2/42), 2.3%% (n=1) edema	PD=25, AEs n=7, 4 died (2 due to PD, 2 due to AEs not related to drug), 1 consent withdrawal	Overall 38% (16/42, 13 G1, 2 G2), G3 n=1 (DLT) as per CTCAE	All managed with premed (NSAIDS, steroids), one patient with G2 received tocilizumab	n=1 discontinued due to G3 CRS, considered a DLT	Neurotoxicity 5% (n=2/42)
NCT03287908/AMG-701 [19]	43% anemia, 23% neutropenia, and 20% thrombocytopenia	-	-	n=58 (PD n=47, AEs 4 (3 CRS, 1 viral pneumonia), consent withdrawal 4, due to other therapy 1, investigator choice 1, CNS disease 1)	Overall 61% (n=45), as G3 6.6% (n=5) per Lee et al.'s criteria	All managed with steroids and tocilizumab	3 discontinued treatment	Neurotoxicity in 8% (n=6) all G 1/2, reversible
NCT03269136/Elranatamab [20]	Anemia, neutropenia, and lymphopenia	-	-	-	Overall 67% at RP2D, all G 1/2, no G 3/4 as per ASTCT	Incidence reduced with premedication; no tocilizumab used	None	None reported
NCT04649359/Elranatamab [21]	Anemia 45.5%, neutropenia 43.1%, thrombocytopenia 26.8%, and lymphopenia 26%	G 3/4 anemia 33.3%, neutropenia 43.1%, thrombocytopenia 20.3%, and lymphopenia 23.6%	Diarrhea 36.6% (n=2), fatigue 2.4% (n=3), hypokalemia 9.8% (n=12), COVID-19 infection 5.7% (7)	Disease progression 32.5%, AE 7.3%	Overall CRS 57.7% (n=71), G 3/4, none. In pts who received priming doses (n=119) CRS was 56.3%, all G 1/2 (n=67) as per ASTCT criteria	44.8% patients (n=30/67), in step-up dose receiving cohort, received tocilizumab and/or steroids	None	17.1% (n=21), G 3/4 0.8%(10/123). In step-up dosage receiving cohort (n=119) 3.4% (n=4) patients had ICANS as per ASTCT criteria
NCT05020236/Elranatamab, Daratumumab [22]	Neutropenia 29%	G 3/4 28%	-	-	50% all G 1/2, as per ASTCT	NA	NA	None seen as per ASTCT
NCT03761108/REGN5458 [23]	-	G3/4 hematologic 39%	Fatigue G 3 in 2 patients (2.7%)	-	28 patients (38.4%), G 1 in 25 patients (34.2%), G 2 in 3 patients (4.1%). No G 3/4	N/A	None	No G≥3 reported
NCT03145181/Teclistamab [24,25]	Neutropenia 65%, anemia 58%, thrombocytopenia 43%, and leukopenia 28%	Neutropenia 48%, anemia 33%, thrombocytopenia 23%, and leukopenia 15%	Diarrhea 2% (n=3), fatigue 1% (n=2), nausea 1% (n=1), cough 1% (n=3), back pain 2% (n=3), arthralgia 2% (n=3), vomiting 1% (n=1)	101/157 patients	57% (89/157) overall (G1 or 2) 70% (28/40) at RP2D, None in G 3/4	Overall 24% (38/157) received tocilizumab, and 15% (23/157) received steroids, in step-up and first full doses	None	Overall 4% (7/157), 1 patient G 1 in RP2D, 1.2% (n=2) patients had G 3/4 in IV dosing, none in SC
	Thrombocytopenia 42%, neutropenia 55%, lymphopenia 40%, and anemia 39%	Thrombocytopenia 29%, neutropenia 50%, lymphopenia 37%, and anemia 29%	Infections G 3/4, 26%	-	63%; all G 1/2	NA	NA	G 3 ICANS in 1 patient, resolved with supportive care and the patient remained on treatment
NCT04722146/SC Teclistamab + Daratumumab + Lenalidomide [26]	Neutropenia 75%	G 3/4 neutropenia 68.8%	Fatigue G 3/4 6.3% (n=2), insomnia G 3/4 3.1% (n=1), infections G 3/4 28.1% (n=9)	-	81.3% (n=26) all G 1/2 as per ASTCT	N/A	N/A	None as per ASTCT
NCT04108195/Teclistamab + Daratumumab [27]	Anemia 46%, neutropenia 54%, and thrombocytopenia 33%	Anemia 28%, neutropenia 54%, and thrombocytopenia 28%	-	-	61%, all G 1/2 as per ATCT	N/A	N/A	1 case G 1 as per ASTCT
NCT03486067/CC-93269/BMS-986349/Alnuctamab/ALNUC [28]	Neutropenia 49%, and anemia 41%, thrombocytopenia 33%	In SC cohorts, neutropenia 30%, and anemia 17%	Infections 47%	17 in SC cohort	Overall 56% (all grades). For IV cohort CRS of 76%	In SC cohorts, n=20 received treatment for CRS (steroids n=8, tocilizumab n=12	None in SC cohorts	-
NCT03933735/TNB-383B/ABBV-383 [29]	Neutropenia 37%, anemia 29%, thrombocytopenia 23%, and lympopenia 15%	G≥3, neutropenia 34%, anemia 16%, thrombocytopenia 12%, and lymphopenia 13%	G ≥3 in overall (n=124) fatigue 1%(1/124), nausea 2% (2/124), diarrhea 2% (2/124)	Overall 64% (79/124); PD 48% (60/124), DLT/AE 6% (7/124). PD is 76% (60/79) of the discontinued patients	All G /≥3g CRS in overall (n=124) 57%(71/124)/2%(3/124), ≥40mg esc+exp cohorts (n=81) 73%(59/81)/4%(3/81), 60mg exp cohort (n=60) patients 71%(36/51)/2%(1/51) as per NCI CTCAE	Managed with common medications or tocilicxumab, resolved completely	In 2% (3/124) patients, CRS lead to dose reduction, no death or d/c due to CRS noted. 14%(n=17) received tocilizumab	1.5% (2/124), both in 60mg cohort

AMG-701

In the first-in-human phase I study (NCT03287908), AMG-701 was administered to 75 patients with RRMM (median age: 63 years). Median disease duration was 5.9 years, and patients of this group had received a median of six therapies previously (Table 1). AMG-701 was given IV weekly in four-week cycles at doses from 5 μg to 12 mg, with a step dosing of 0.8 mg (Table 2) [19].

At doses 3-12 mg, ORR was 36% (n=16/45), including four sCR (three MRD-negative, one pending), one MRD-negative CR, six VGPR, and six PR. At ≤1.6 mg, only one patient responded. In the cohort of patients receiving 9 mg, ORR was 83% (5/6; 3 PR, two VGPR). Median response time was one month, and median DoR was 3.8 months, with a maximum duration of 23 months; 14 of 17 responses were ongoing at cutoff. All four patients who showed MRD achieved negativity, which was sustained for up to 20 months (Table 3) [19].

CRS occurred in 61% of patients, resolving with corticosteroids/tocilizumab. Other DLTs included atrial fibrillation, acidosis, and thrombocytopenia. AEs related to the blood included anemia (43%), neutropenia (23%), and thrombocytopenia (20%). Neurotoxicity occurred in six patients, all grades 1-2, and was reversible. Serious AEs occurred in 39% (n=29), most commonly infections (n=13) and CRS (n=7). Four deaths occurred, none treatment-related (Table 4) [19]. AMG-701 showed manageable safety and encouraging anti-myeloma activity at higher doses, supporting further clinical development [19].

Elranatamab (MagnetisMM-1)

In the ongoing first-in-human phase I trial (NCT03269136), elranatamab, a bsAbs targeting BCMA and CD3, was administered subcutaneously to 55 patients with RRMM (median age: 64 years). Patients had received a median of five prior regimens (Table 1) [20]. Elranatamab was given either weekly or biweekly at a dose of 80-1000 μg/kg subcutaneously (Table 2). PK analyses showed dose-dependent exposure; soluble BCMA decreases with disease response, and elranatamab induced T-cell proliferation [20].

Median response time was 36 days. At a median follow-up of 12.0 months, ORR was 64%. However, in patients with prior BCMA-directed therapy, ORR was 54% (7/13) (median DoR: 17.1 months). All evaluable patients with CR or better (12/12) achieved MRD negativity at 10^-5^ sensitivity, including two with ongoing MRD-negative stringent CR beyond two years (Table 3) [20].

With pre-medication and a single priming dose (600µg/kg or 44mg), CRS occurred in 67% of patients. Additional common adverse effects included injection site reactions, lymphopenia, neutropenia, and anemia (Table 4). Elranatamab demonstrated a potentially manageable and safe profile with durable efficacy in heavily pretreated RRMM, supporting further development in pivotal trials.

Elranatamab (MagnetisMM-3)

In MagnetisMM-3, elranatamab monotherapy was evaluated in 123 patients with RRMM previously treated with BCMA-directed therapies. Median age was 68.0 years (range 36-89), and 55.3% of them were males. A total of 63.4% of the patients had ECOG ≥1; 25.2% had high-risk cytogenetics, and 31.7% had extramedullary disease. Patients had received a median of five prior lines of therapy (range 2-22). Most were refractory to therapy: 96.7% triple-class and 42.3% penta-drug (Table 1) [21].

Elranatamab 76 mg was given subcutaneously once a week on a 28-day cycle with a two-step-up priming dose regimen (12 mg and 32 mg) administered during the first week in MagnetisMM-3. Median follow-up was 6.8 months, with a median treatment duration of 5.3 months; 51.2% remained on therapy (Table 2). The most common reason for discontinuation of treatment was progression of disease (32.5%) (Table 4) [21].

ORR was 61.0% (95% CI, 51.8-69.6). Median time to response was 1.2 months (range, 0.9-6.9). Median DoR was not reached. Median PFS was noted as 90.4% (95% CI, 79.8-95.6) at six months; median OS was not achieved (Table 3) [21].

The most common treatment-emergent adverse events (TEAEs) were infections (61.8% grade 3/4, 31.7%), CRS (56.3% in patients who received priming doses), anemia (45.5%, grade 3/4 33.3%), neutropenia (43.1%, all grade 3/4), thrombocytopenia (26.8%, grade 3/4 20.3%), diarrhea (36.6%), lymphopenia (26%, grade 3/4 26.3%) and peripheral neuropathy (17.1%). ICANS occurred in 3.4% (Table 4) [21]. Elranatamab demonstrated clinically meaningful activity with durable responses and a manageable safety profile in RRMM patients previously exposed to BCMA-directed therapies.

Elranatamab + DARA (MagnetisMM-5, Part 1)

In the open-label phase III MagnetisMM-5 trial (NCT05020236), a safety lead-in evaluated subcutaneous elranatamab plus subcutaneous DARA in patients with RRMM. Eligible patients had received ≥3 prior lines, including lenalidomide and a proteasome inhibitor; prior BCMA-targeted therapy was excluded (Table 1). Elranatamab was administered with a two-step priming regimen during cycle 1, followed by full weekly dosing through cycle 6 and every two weeks thereafter in responders. DARA was given per standard prescribing information (Table 2) [22].

As of April 2022, 28 patients were treated. Median age was 68 years (range 46-78), median prior regimens were five, 18% were triple-class refractory, and 71% had prior autologous SCT. Median treatment duration was 6.8 weeks (0.1-23.1) (Table 1). Most patients (93%) experienced at minimum of one TEAE, and 46% were noted to have grade 3/4 TEAEs. The most common events were CRS (50%; all grades 1-2), neutropenia (29%; 14% grade 3, 14% grade 4), and pyrexia (21%; all grade 1). CRS typically occurred after the first elranatamab dose, with a median onset of two days (range 1-4); events led to dose interruption in 7% but no permanent discontinuations. No ICANS or DLTs were observed (Table 4) [22]. Promising early activity was noted, including VGPR and sCR. Median time to response was one month (range 1-3) (Table 3) [22].

Elranatamab combined with DARA showed manageable safety with encouraging efficacy signals, consistent with prior monotherapy results. These findings support continued development, with part 2 of MagnetisMM-5 randomizing patients 1:1:1 to elranatamab monotherapy, elranatamab plus DARA, or DARA plus pomalidomide/dexamethasone [22].

Linvoseltamab (REGN5458)

In the first-in-human phase I/II trial (NCT03761108), REGN5458, a bsAbs targeting BCMA and CD3, was evaluated in 73 patients (median age: 64 years; median prior lines of therapy: 5) with RRMM. The RISS stage was I, II, or III in 15%, 60%, and 24%, respectively (Table 1). REGN5458 was administered IV weekly for four months, then biweekly, at doses from 3 to 800 mg. Median follow-up was 3.0 months (range, 0.7-22.1) (Table 2) [23].

Responses were observed at all doses. ORR was 50.6% (37/73). Across all cohorts, 86.5% of those who responded displayed VGPR, and 43.2% achieved CR. Patient cohorts treated at 200-800mg dosage had an ORR of 75.0% (n=18/24). The estimated median DoR at eight months was 90.2% (95% CI 72.6-96.7%). Responses were independent of BCMA expression level (Table 3) [23].

The most common TEAEs were fatigue (45.2%), CRS (38.4%, grade 1 in 34.2%, grade 2 in 4.1%), with no grade ≥3 events and no discontinuations, pyrexia (35.6%), and nausea (32.9%). Grades 3 and 4 TEAEs were reported in 42.5% and 32.9%, respectively. The most common grade 3/4 TEAEs were hematologic (39.0%) (Table 4). A consistent safety profile was observed across all the dose levels, demonstrating no correlation between CRS and dose [23]. Linvoseltamab demonstrated an acceptable safety profile with only low-grade CRS and no neurotoxicity, and it induced deep, durable responses in heavily pretreated, triple- and penta-refractory RRMM. Phase II evaluation is ongoing.

TEC (MajesTEC-1)

In the open-label phase I MajesTEC-1 study (NCT03145181), TEC (IV: 0.3-720 μg/kg or SC: 80-3000 μg/kg), a bsAbs targeting BCMA and CD3, was given to 157 RRMM patients (median prior therapies: 6) (Table 1). Routes of administration were either IV or SC with step-up dosing for ≥38.4 μg/kg. At the RP2D, 40 patients were treated (median follow-up 6.1 months) at a dose of SC 1500 μg/kg weekly after 60 and 300 μg/kg step-up doses (Table 2) [24]. 

Among response-evaluable patients at the RP2D (n=40), ORR was 65% (95% CI 48-79), with 58% achieving VGPR or better. Median response duration was not reached; at 7.1 months’ median follow-up, 85% of those responders remained alive and on treatment. TEC maintained exposure above target levels and induced consistent T-cell activation (Table 3) [24]. No DLTs were observed. At RP2D, 70% of patients had CRS (28/40, all grades 1-2), and 65% (26/40) had neutropenia. Other AEs were consistent with immunotherapy-related toxicities (Table 4) [24].

In cohort C (Touzeau et al.) of the MajesTec-1 trial, 25/38 (66%) RRMM patients with prior exposure to anti-BCMA therapy were recruited (Table 1). Patients received SC TEC weekly at a 1.5 mg/kg dose (Table 2). The ORR was 40% (95% CI, 21-61) at a median follow-up of 6.9 months (range 0.7-8.7). CR or better was observed in five patients (20%). TEAEs included 63% CRS (all G1/2); infections 42% (16/38; G3/4, 26%); neutropenia 55% (G3/4, 50%); thrombocytopenia 42% (G3/4, 29%), and lymphopenia 40% (G3/4, 37%) (Table 4) [25]. TEC demonstrated a favorable safety profile and deep, durable responses in heavily pretreated RRMM, supporting further clinical development.

TEC + DARA + Lenalidomide (MajesTEC-2)

In the open-label phase I/II MajesTEC-2 trial (NCT04722146), TEC (0.72 mg/kg or 1.5 mg/kg) was evaluated in combination with DARA (1800 mg) and lenalidomide (25 mg) (TEC-DARA-LEN) in 32 patients (median age: 62 years; 88% Male) with MM who had received a median of two prior lines of therapies (range 1-3). A total of 19% were DARA-refractory, and 28% lenalidomide-refractory (Table 1). The regimen was administered weekly (Table 2) [26].

Efficacy was promising. The overall ORR was 89.65% (n=26/29), 100% (13/13) in the 0.72 mg/kg cohort, and 81.25% (n=13/16) in the 1.5 mg/kg cohort. VGPR was 92.3% (12/13) for the 0.72 mg/kg cohort, but did not reach for the 1.5 mg/kg group. Median response time was one month. Pharmacokinetic (PK) and cytokine profiles were comparable to TEC monotherapy, with evidence of T-cell activation (Table 3) [26].

Patients were followed up for a median of 5.8 months (median treatment: five months). CRS was the most common AE, reported in 81.3% (n=26), all grades 1/2, mostly during cycle 1 (median onset two days, median duration two days). No ICANS occurred. Other frequent AEs included neutropenia (75%), fatigue (44%), diarrhea (38%), insomnia (31%), cough (28%), hypophosphatemia (25%), pyrexia (25%), and febrile neutropenia (13%). Infections occurred in 75% (n=24), with grades 3/4 in 28%, most commonly upper respiratory infection, COVID-19, and pneumonia (each 22%). One patient (3%) discontinued and died due to COVID-19 (Table 4) [26]. TEC-DARA-LEN demonstrated a manageable safety profile consistent with the individual agents and showed high early response rates, supporting phase III evaluation in the MajesTEC-7 trial in transplant-ineligible newly diagnosed MM.

TEC + DARA (TRIMM-2)

In the phase Ib TRIMM-2 study (NCT04108195), TEC, a BCMA×CD3 bsAbs, was combined with DARA, a CD38 monoclonal antibody. Eligible RRMM candidates who had received ≥3 previous lines, or were double-refractory, had prior anti-CD38 therapy within 90 days, were excluded (Table 1). TEC (1.5-3 mg/kg) was given SC weekly or biweekly with step-up dosing; DARA was administered SC 1800 mg per standard schedule (Table 2) [27]. At data cutoff (January 2022), 46 patients were treated with a median age of 67 years and had received a median of six prior lines (Table 1). Median follow-up was 7.2 months (0.1-16.6) [27].

Thirty-seven patients were evaluated, and the ORR was 78% (29/37), with VGPR or better in 73% (27/37) and CR or better in 24.3% (9/37). Median time to response was one month. Across cohorts, ORR was 75% (15/20) at TEC 1.5 mg/kg weekly, 100% (4/4) at TEC 3 mg/kg weekly, and 77% (10/13) at TEC 3 mg/kg every two weeks (Table 3) [27].

AEs occurred in 91% of patients, grade 3/4 in 78%. CRS was the most frequent (61%, all grade 1-2; median onset two days, duration two days). Other common events were neutropenia (54%), anemia (46%), thrombocytopenia (33%), and diarrhea (33%). Infections occurred in 63% (n=29), grade 3/4 in 28%. However, grade 1 ICANS developed in one patient, which was resolved (Table 4) [27]. Finally, TEC + DARA showed a safety profile that was manageable clinically, with no overlapping toxicities; however, it showed promising efficacy in heavily pretreated RRMM, supporting the potential synergy of dual immunotherapy.

Alnuctamab (ALNUC)

In the open-label phase I study (NCT03486067), ALNUC, a 2+1 BCMA×CD3 T-cell engager, was administered to patients with RRMM. Patients had received ≥3 prior lines (Table 1). Both IV and SC administration were assessed [28]. Seventy patients received IV ALNUC at doses of 0.15-10 mg (Table 2). ORR was 39% (27/70) with a median DoR of 33.6 months. Median PFS was 3.1 months (Table 3). CRS occurred in 76% (53/70) (Table 4) [28]. Seventy-three patients (median age: 64 years; median prior regimens: 4) received SC ALNUC in escalation (10-60 mg) or expansion cohorts (10-60 mg). Median follow-up was 4.3 months; 53% remained on treatment (Table 2) [28].

An ORR of 53% was seen across all doses and 65% at the 30 mg dose among all 55 efficacy-evaluable SC patients. Sixteen of the 20 evaluable responders (80%) achieved MRD negativity (10^-5^ by flow cytometry). Median time to response was 1.0 month (95% CI 0.7-1.3), of which 86% of responses were ongoing. PK analysis showed ~61% bioavailability and a 15-day half-life, with drug levels at 30 mg exceeding predicted efficacy thresholds (Table 3) [28].

AEs occurred in 99% of patients, CRS (56%), neutropenia (49%), infections (47%), anemia (41%), and thrombocytopenia (33%). CRS was low-grade, with a median onset of three days and a median duration of two days; 30% required tocilizumab and 15% corticosteroids. Two patients developed grade 1 neurotoxicity. One patient discontinued due to metastatic colon cancer. And one death occurred due to cerebral hemorrhage at 60 mg and was related to the treatment (Table 4) [28]. IV ALNUC demonstrated durable responses but high CRS incidence, whereas SC ALNUC improved tolerability with dose-dependent activity, high MRD negativity rates, and manageable safety. The study is ongoing.

ABBV-383 (TNB-383B)

In the phase I study (NCT03933735), ABBV-383, a BCMA×CD3 bsAbs, was evaluated in 124 patients with RRMM (median age: 68 years; 55% male). Fifty-seven percent of them had ECOG PS 1 with a median of five prior lines (Table 1). At cutoff (January 2022), 36% remained on treatment; 64% discontinued, mostly due to progression (76%) [29].

In efficacy-evaluable patients (n=122), ORR was 57% overall and 59% in the 60 mg cohort, with ≥VGPR in 43% overall. In ≥40 mg cohorts (n=79), ORR was 68% and ≥VGPR 54%. Among 11 MRD-evaluable patients with CR/sCR, 73% were MRD-negative. In triple-class refractory patients (n=100), ORR was 51% overall, 63% in ≥40 mg cohorts, and 54% in the 60 mg cohort. In high-risk cytogenetics (n=11, ≥40 mg cohorts), ORR was 82% with 27% CR/sCR. Median DoR was not reached; six- and 12-month DoR rates were ~75-80%. Median PFS was not reached in ≥40 mg and 60 mg cohorts; overall PFS was 10.4 months (Table 3) [29].

AEs occurred in 98% of patients. The most common hematological adverse effects were neutropenia (37%) and anemia (29%). Common non-hematologic events included CRS (57%), fatigue (30%), nausea (29%), and diarrhea (27%). In cohorts with a dosage of ≥40 mg, CRS occurred in 73% (all grades). Median onset was one day, and median duration was one day; 14% received tocilizumab. Serious TEAEs occurred in 53%, most commonly infections (41%; grade ≥3 in 25%). ICANS was reported in two patients at 60 mg. TEAEs led to discontinuation in 10%, interruption in 37%, and dose reduction in 5%. Seven patients (6%) died due to TEAEs (COVID-19, sepsis, liver injury, subdural hematoma, myeloma), none of which were considered treatment-related (Table 4). The maximum tolerated dose (MTD) was not reached; 60 mg Q3W was selected for expansion [29]. ABBV-383 demonstrated durable responses, was manageable and safe at 60 mg Q3W, supporting continued development in heavily pretreated RRMM.

Discussion

In early, single-arm trials of anti-BCMA bsAbs, reported ORRs have varied between 25% with AMG-701 [19] and 70% with TEC [24] and ALNUC [28]. ABBV-383 [29] and REGN5458 [23] have also shown promising activity in the 50-65% range. These outcomes must be interpreted with caution, since the trial populations, exposure to previous therapy, refractory state, and the duration of follow-up varied significantly across studies. This data indicates a potentially promising efficacy with the use of BCMA bispecifics in the heavily pretreated RRMM. In patients with disease refractory to prior anti-BCMA therapy, ORR for TEC and elranatamab was 40% and 54%, respectively [20,24]. Similarly, trials using bsAbs in combination with lenalidomide or DARA have demonstrated high response rates and an additive or synergistic effect [22,26,27]. AMG-420 was withdrawn from the clinical trials due to its frequent need for transfusions owing to the short half-life [18]. Moreover, anti-BCMA bsAbs have been associated with overall manageable safety profiles, the majority of which are low-grade and reversible. Although AEs were rare, which led to treatment discontinuation, care must be taken before concluding because of the variations in study design, patients, and follow-up periods. Among these, the incidence of overall and G3/4 hematological toxicity (anemia, neutropenia, thrombocytopenia, lymphopenia) was high with TEC, followed by elranatamab, ALNUC, and ABBV-383 [20,24,28,29]. Infections (G1-5) were a relatively common side effect of these drugs, and were more frequently seen with TEC, ABBV383, AMG-420, and elranatamab, which warrants proper antibiotic prophylaxis and monitoring of patients with comorbid conditions, given that MM causes immunocompromised status [18,20,24,29]. The incidence of CRS was high in the trials (ranging from 38% to 81.3%); however, most cases were G1/2, and only a few were G3/4. Most cases were manageable with paracetamol and steroids in a majority of cases, and sometimes it necessitated the use of tocilizumab [18,19,28,29]. Its incidence was reduced significantly with SC rather than IV administration of the drug, use of priming/step-up doses, and/or premedication with steroids [20,21,24,28]. It led to discontinuation of treatment in four patients (AMG420, AMG 701) and dose reduction in three patients (ABBV383) across the trials; noticeably, there was no death recorded due to CRS [18,19,29]. The occurrence of immune effector-cell-associated neurotoxicity syndrome did not lead to any treatment alteration. Early, drastic, and sustained reduction in soluble BCMA level was observed, and its use to predict response to treatment was supported (elranatamab, AMG 420, AMG701) [18-20]. Moreover, antibodies to anti-BCMA-CD3 bsAbs were rarely reported.

Anti-BCMA CAR-T cell therapies and ADCs have been approved for clinical use. Many of the anti-BCMA-CD3 bsAbs have similar or improved safety and efficacy outcomes compared to ADCs and CAR-T cell therapy. Belantamab mafodotin in DREAMM-1/2 trial showed an ORR of 60%/30-34% with incidence of corneal toxicity of 53-63%/21-27%, G 3/4 thrombocytopenia 34%/20-33%, and anemia 14%/20-25% (all G3/4) [30,31]. Idecabtagene vicleucel had an ORR of 73%, CRS 84% (mostly G1/2), and neurotoxicity in 18% participants in the KarMMa trial [32]. CARTITUDE 1/2 trials revealed an ORR of 97%/95%, G3/4 cytopenias of 50-95%/35-95%, and CRS incidence 95%/95% (predominantly G1/2) for ciltacabtagene autoleucel [33,34]. In comparison to these findings, bsAbs have a favorable efficacy (ORR 25-90%) and safety profile (CRS 38-81%). Additionally, off-the-shelf availability makes these drugs more preferable.

Our approach was limited in certain areas. The trials included were mostly early-phase trials with short follow-ups, small sample sizes, and non-homogeneous populations. They were mostly single-arm designs devoid of randomization and control groups, which restricted the power of inference. Reported outcomes were descriptive, with few statistical comparisons across subsets, and there was wide variation in eligibility criteria, previous lines of therapy, refractory status, and definitions of response or follow-up duration. Such considerations exclude direct cross-trial comparisons of efficacy and safety, and relative activity between various agents or regimens should be interpreted with caution.

Anti-BCMA bsAbs are a promising emerging therapeutic class in RRMM. Despite these limitations, durable responses in patients with extensive prior treatment, and with manageable safety profiles, have been observed. A number of agents have shown promising activity; TEC, elranatamab, ALNUC, linvoseltamab, and ABBV-383 have all demonstrated encouraging activity, and initial combination studies are encouraging the possibility of additive or synergistic effects when combined with existing DARA or lenalidomide. These observations, however, are exploratory and hypothesis-generating as opposed to confirmatory.

Future studies should fill this gap via randomized controlled trials with larger sample sizes, standardized endpoints, and longer follow-ups to establish the actual survival advantage, response-sustained durability, and relative safety of such agents. Also, long-term follow-up is needed to identify late or cumulative toxicities, risk of infection, and effects of sequential BCMA-targeted therapy.

## Conclusions

This systematic review showed that bsAbs were effective in managing RRMM. The main side effects observed were cytokine release syndrome and blood-related toxicities, which were typically mild and controllable. Owing to their strong therapeutic potential and acceptable safety profile, BCMA-directed bsAbs are becoming an important new treatment approach for patients with advanced or difficult-to-treat MM. In conclusion, RRMM can be managed by a novel immunotherapeutic approach using the BCMA-targeted bsAbs, but conclusive evidence of their optimal place in treatment sequencing, comparative efficacy, and long-term safety will need strict phase III trials.
